# Adenosine Deaminase (ADA) Level in Tubercular Pleural Effusion

**DOI:** 10.4103/0970-2113.44121

**Published:** 2008

**Authors:** S.K. Verma, A.L. Dubey, P.A. Singh, S.L. Tewerson, Davashish Sharma

**Affiliations:** 1 Department of TB & Chest Diseases, CSM Medical University, Lucknow, India; 2 Department of Pathology, CSM Medical University, Lucknow; M.L.N. Medical College, Allahabad, India; 3 Department of Statistics, CSM Medical University, Lucknow; M.L.N. Medical College, Allahabad, India

**Keywords:** Tubercular Pleural effusion, Pleural fluid ADA level

## Abstract

**STUDY OBJECTIVE:**

To study the value of adenosine deaminase level in tuber-cular pleural effusion.

**DESIGN:**

A hospital based observational study.

**SETTING:**

Out and In patients service of department of Tuberculosis & Chest Diseases, MLN Medical College Allahabad.

**PATIENTS:**

50 consecutive patients of pleural effusion, who were above the age of 12 years, were studied.

**RESULTS:**

Pleural fluid adenosine deaminase was more than 36 IU/L (36 to 229.7 IU/L) in tubercular pleural effusion (34 patients). In case of malignancy no. of patients was 08 and pleural fluid adenosine deaminase was more than 18.5 IU/L (18.5 to 87.6 IU/L). While in one case of hypoprotenemiea pleural fluid adenosine deaminase was 8.21 IU/L. If 36 IU/L is taken as cut of limit the sensitiv-ity and specificity of ADA for tuberculosis is 100 % and 77.7 %. More than 100 IU/L was exclusively seen in tubercular pleural effusion.

**CONCLUSION:**

ADA > 100IU/L was observed in TB only.

## INTRODUCTION

Pleural effusion is a common chest problem, yet it is difficult to establish the aetiological diagnosis in as many as 20% cases in spite of good history, thorough clinical, radiological, full examination of aspirated fluid and pleural biopsy^1^. So there is a need of simple, rapid and reliable diagnostic test to establish the aetiology of pleural effusion. Considering this a prospective hospital based study was designed to compare pleural fluid adenosine deaminase level and pleural biopsy in establishing the diagnosis of tubercular pleural effusion.

## MATERIAL AND METHOD

The study comprised of 50 consecutive patients of pleural effusion, both male and female, above the age of 12 years who attended the Swaroop Rani Nehru Hospital of Motilal Nehru Medical College, Allahabad, U.P. India. Patients in whom history of typhoid fever, acute viral hepatitis and active cirrhosis were present, were excluded. Detailed history was taken and thorough clinical examination was done in each and every patients and they were then subjected to a batteries of investigation which included routine haemogram, urine examination, skiagram chest PA and lateral view, sputum smear examination for AFB and sputum culture for mycobacterium tuberculosis, pleural fluid for protein, glucose, cell count, malignant cells, Gram's stain, pleural fluid examination for AFB, pleural fluid culture for mycobacterium tuberculosis and other relevant investigation as per need of cases. ADA was measured in pleural fluid by colorimetric method of Guisti and Galanti^2^. Pleural biopsy was done through Abraham's punch biopsy needle^3^.

## RESULTS

50 patients above the age of 12 years were studied. Male were 34 and female were 16.

Out of 50 patients tuberculosis was diagnosed in 34 cases (by history + sputum results + pleural fluid results +pleural biopsy); 19 (46%) patients were diagnosed by pleural biopsy; 11 were diagnosed by AFB in pleural fluid and 4 by AFB in sputum smear examination. Similarly 8 cases of malignancy were diagnosed (4 by direct histology of pleural tissue and 4 by tissue biopsy from lung parenchyma mass or lymph node). Pleural fluid ADA level was more than 36 IU/L in cases of tubercular pleural effusion. It ranged 36 to 229.7 IU/L. While in case of malignancy it was more than 18.5 IU/L (18.5 to 87.6 IU/L). In one case of hypoproteinemia it was 9.21 IU/L. When 36 IU/L is taken as cut off point, sensitivity and specificity of ADA for TB is 100% and 77.7%. We found that when more than 100 IU/l was taken as cut of limit of ADA level, it was seen in tuberculosis only.

## DISCUSSION

Present study confirms that t ADA level in tubercular pleural effusion is increased and in non tubercular pleural effusion ADA level did not exceed to100 IU/L. Tuberculosis is a common cause of pleural effusion^4^ especially in countries like India. More over incidence of tuberculosis is increasing world wide^5^. Although tubercular pleural effusion can resolve spontaneously but up to 65% untreated tubercular pleural effusion can develop active tuberculosis^6^. So rapid and accurate diagnosis and prompt treatment is necessary for tubercular pleural effusion. Whenever a patient of pleural effusion presents we usually investigate online of gross, microscopic and biochemical parameters (excluding ADA level). Although lymphocytic predominant fluid is usually seen in tubercular pleural effusion but all lymphocytic predominant fluid can't be tubercular, it could be malignant. So there is a need to differentiate among various causes of pleural effusion. Definitive diagnosis of tubercular is often difficult as in more than 50% of patients, pleura is the only site of infection^7^. Tuberculin test is non specific and finding can be negative^8^. Because bacterial load is less^9^ so pleural fluid culture for mycobacterium tuberculosis is also low (< 20) 10. Pleural fluid ADA estimation is quick and relatively inexpensive.

In present study ADA level in tuberculosis cases was more than 36 IU/L in agreement with Niwa et al. (1985)^11^ >38IU/L; Rodziguez (1962)^12^ >37 U/L and Jindal et al(1993)^13^ > 40U/l. In case of malignant pleural effusion our findings co- relate with most of the authors. ADA level in malignancy was up to 87.6 IU/L. ADA level more than 100 IU/L observed only in cases of tubercular pleural effusion so from the study we concluded that if ADA level of more than 100 IU/L is taken as cut off point it is exclusively seen in cases of tubercular pleural effusion. So we can say that estimation of ADA level in pleural fluid is extremely helpful in establishing the aetiology of tubercular pleural effusion and to rule out other diagnosis especially of other diseases in which lymphocyte predominance of pleural effusion is seen such as malignancy and collagen vascular diseases (i.e. rheumatoid arthritis and systemic erythematosus).

### Limitation of study :

Number of patients studied is small. So definitive criteria can't be established on this sample size. A large number of patients are required to confirm our findings further and establish the definitive criteria.
